# Quorum sensing in *Porphyromonas gingivalis* and oral microbial interactions: a scoping review

**DOI:** 10.3389/froh.2025.1573863

**Published:** 2025-06-06

**Authors:** Zelda Ziyi Zhao, Wenwen Shan, Lifeng Guo, Chun Hung Chu, Jing Zhang

**Affiliations:** ^1^ Faculty of Dentistry, The University of Hong Kong, Hong Kong, Hong Kong SAR, China; ^2^ Key Laboratory of Oral Diseases Research of Anhui Province, College & Hospital of Stomatology, Anhui Medical University, Hefei, China

**Keywords:** quorum sensing, microbial interaction, *Porphyromonas gingivalis*, biofilms, periodontal disease

## Abstract

**Objective:**

*Porphyromonas gingivalis*, a major periodontal pathogen, interacts with other oral microbes through quorum sensing, enhancing its growth and virulence, which contributes to periodontitis. This scoping review aims to examine the role of quorum sensing in the interactions between *P. gingivalis* and other oral microbial species.

**Methods:**

Two independent researchers conducted a systematic search using the keywords {[(quorum sensing) OR QS] AND [(*Porphyromonas gingivalis*) OR *P. gingivalis*]} for English publications prior to 2025 from Medline, Scopus, and Web of Science databases. They screened titles and abstracts, retrieving full texts of original studies to identify key concepts and findings regarding the quorum sensing of *P. gingivalis* in oral microbial ecosystems.

**Results:**

A total of 205 publications were identified, of which 26 were included in the review. These studies demonstrated quorum sensing of *P. gingivalis* and other bacteria through signal molecules Autoinducer-1 and Autoinducer-2. Autoinducer-1 enhances the pathogenicity of *P. gingivalis*, facilitating its integration into complex oral microbial communities. Autoinducer-2 fosters cooperative or competitive relationships between *P. gingivalis* and other periodontal pathogens, modifying the structure of oral biofilms. Additionally, researchers are studying the use of quorum sensing inhibitors to reduce the virulence of *P. gingivalis* for managing periodontitis and restoring microbial balance in the oral cavity.

**Conclusion:**

Quorum sensing enhances the pathogenicity of *P. gingivalis* in the oral environment. Through the modulation of Autoinducer-1 and Autoinducer-2, quorum sensing regulates interactions between *P. gingivalis* and other oral microbes. This study demonstrates the need for further research into quorum sensing-targeted interventions in periodontal therapy.

## 1 Introduction

Oral cavity is a highly complex and dynamic microbiome, consisting of diverse bacterial species that coexist and engage in complex interactions (1). *Porphyromonas gingivalis* (*P. gingivalis*) stands out among these as a clinically significant gram-negative anaerobe, primarily found in biofilms on the surfaces of teeth and soft tissues. The interactions of *P. gingivalis* with other microorganisms are of particular interest because they are implicated in disrupting the homeostasis (the stable balance of microorganisms and conditions in the oral environment) and are crucial in the pathogenesis of periodontal disease, a prevalent chronic inflammatory condition (2).

Bacteria rely on quorum sensing a sophisticated mechanism to perceive and respond to changes in cell-population density through the production and detection of signaling molecules (3). When bacterial populations reach a critical density, this regulatory system triggers coordinated changes in gene expression and physiological behavior, such as virulence factors expression, biofilm formation, survival and colonization (4). Quorum sensing mechanisms are commonly studied using methodologies, including molecular detection techniques such as LC-MS/MS for quantitative analysis of autoinducers and reporter strain assays (e.g., *Vibrio harveyi*) for functional validation, genetic approaches such as *LuxS/luxR* gene knockout, omics technologies such as RNA-seq transcriptomics and mass spectrometry-based metabolomics, and *in vitro* biofilm models. In the context of oral microbiology, quorum sensing emerges as a significant mechanism influencing the pathogenic traits of *P. gingivalis*, enhancing its ability to form resilient and complex biofilms and to engage in interspecies interactions that can affect the overall microbial community structure (5, 6). Two quorum sensing systems are involved in *P. gingivalis* (3). The Autoinducer-1 (AI-1) system predominantly regulates intraspecies communication. Recent studies suggest that AI-1 also plays a role in facilitating interactions between *P. gingivalis* and other species, such as *Streptococcus mutans* (*S*. *mutans*), within mixed-species biofilms, thus highlighting the nuanced roles AI-1 plays in both maintaining species-specific networks and mediating broader ecological interactions (7). On the other hand, Autoinducer-2 (AI-2) serves as a universal signal molecule involved in interspecies communication, bridging both gram-positive and gram-negative bacteria (8). This signaling capability allows *P. gingivalis* to engage synergistically with various pathogens, thereby exacerbating the severity of periodontal diseases and complicating the microbial landscape of the oral cavity. The modulation of its interactions through AI-1 and AI-2 not only impacts its own pathogenicity but also significantly alters the ecological balance of the oral microbial community.

**Figure 1:**
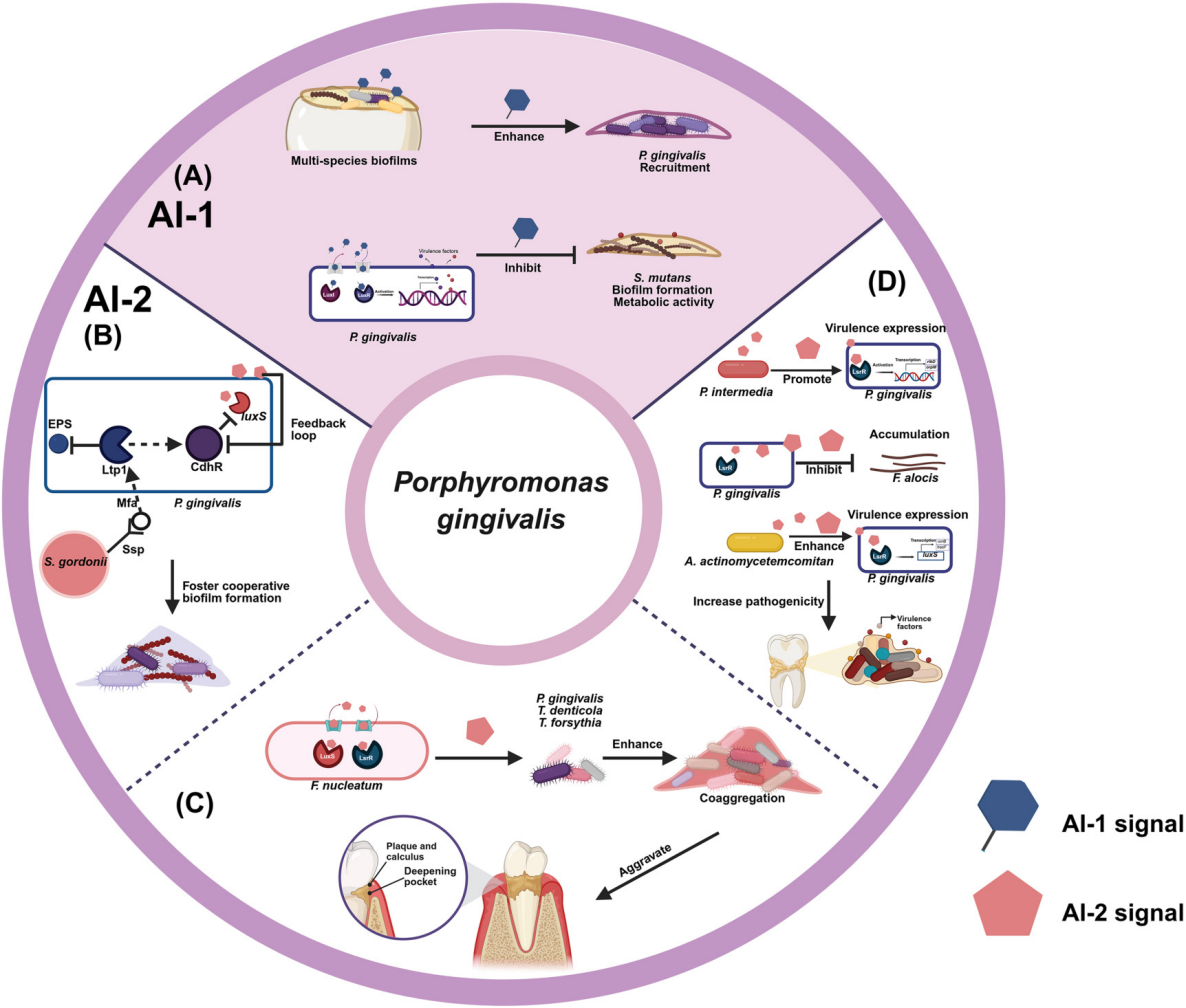
Diagrams of quorum sensing in *P. gingivalis*. **(A)** Modulation of *P. gingivalis* interactions with other bacterial species by AI-1 **(B)** commensality of *P. gingivalis* interactions with *S. gordonii* by AI-2 **(C)** promotion of *P. gingivalis* interactions with *F. nucleatum* by AI-2 **(D)** collaboration of *P. gingivalis* interactions with other bacterial species by AI-2. Created in BioRender. Zhao, Z. (2025) https://BioRender.com/o95s786.

Considering the critical role of quorum sensing in influencing both cooperative and competitive interactions of *P. gingivalis* with other microbial species in oral, there is increasing interest in strategically targeting quorum sensing mechanisms using inhibitors. These inhibitors disrupt the intercellular communication pathways mediated by quorum sensing, thereby representing a potential therapeutic approach for combating *P. gingivalis*-associated diseases. Despite the burgeoning literature on quorum sensing and its implications in *P. gingivalis*, a cohesive understanding of how quorum sensing regulate microbial interactions with *P. gingivalis* remains limited. Therefore, this scoping review aims to elucidate the role of quorum sensing in regulating the interactions between *Porphyromonas gingivalis* and other oral microbial species. Ultimately, this review will provide insights into the potential of targeting quorum sensing pathways as a means to combat periodontal disease and enhance overall oral health (Figure 1).

## 2 Methods

**Figure 2:**
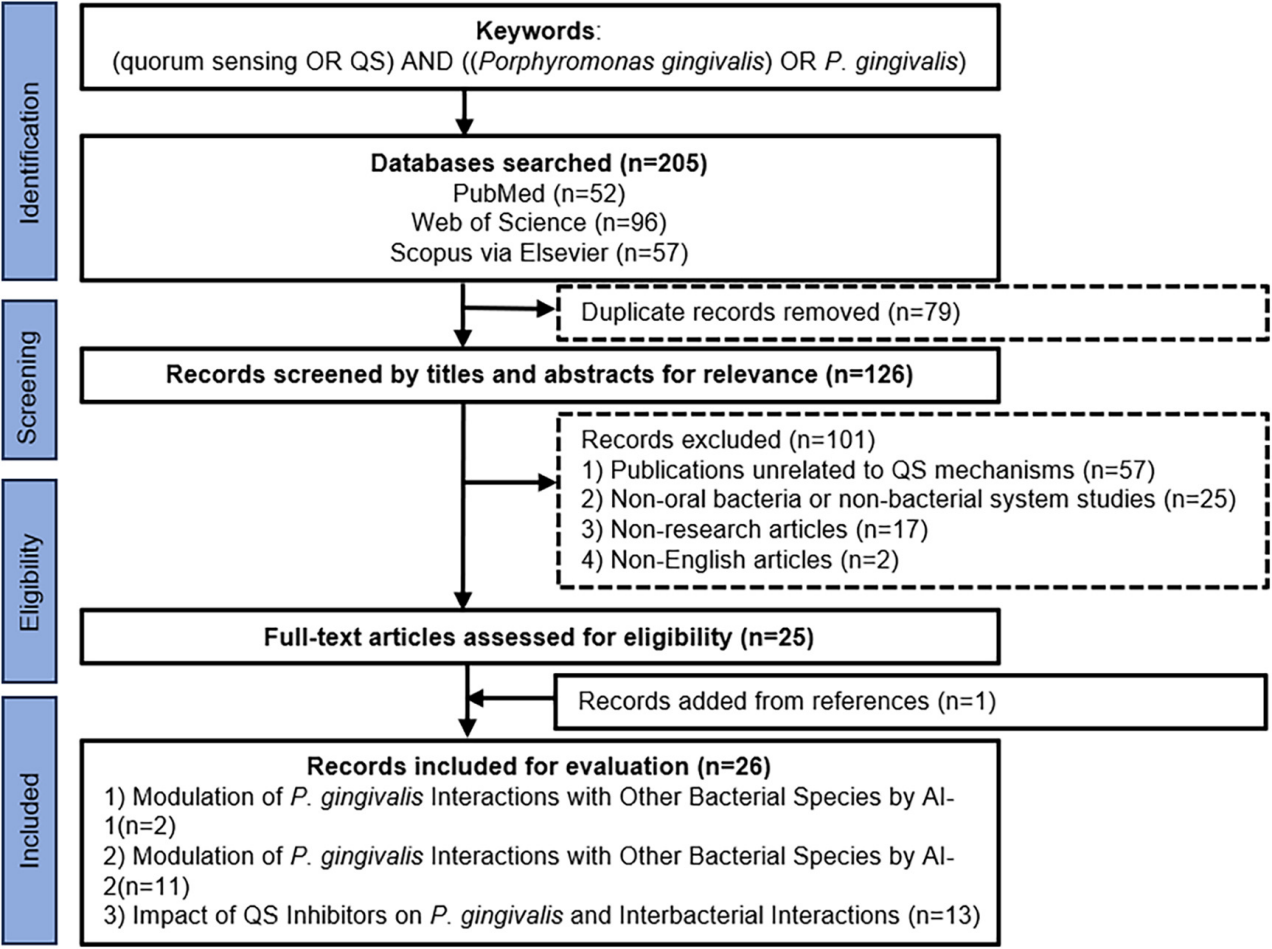
Flowchart of the literature search.

This scoping review followed Arksey & O'Malley's framework and PRISMA-ScR guidelines. To avoid selection biases, a comprehensive search strategy with explicit methodology and transparent reporting was conducted. Two independent researchers systematically searched three databases (PubMed, Web of Science and Scopus via Elsevier) for English-language articles published prior to 31 Dec 2024. The keywords used for relevant articles were “(quorum sensing OR QS) AND [(*Porphyromonas gingivalis*) OR *P. gingivalis*]”. Eligibility criteria were defined as: (i) population: studies investigating *P. gingivalis* and its interactions within oral microbial communities; (ii) concept: studies focusing on quorum sensing mechanisms or quorum sensing inhibitors; (iii) context: oral microbiome studies; (iv) study types: *in vitro* experiments, animal models, clinical trials or human observational studies; (v) language/date: English-language publications prior to 31 December 2024. Exclusion criteria included: (i) studies lacking direct investigation of quorum sensing mechanisms; (ii) studies focusing on non-oral bacteria or non-bacterial systems; (iii) non-research articles, e.g., reviews without novel data, editorials, conference abstracts. A complete PRISMA-ScR flow diagram showed the screening process and reasons for exclusion at each stage. From 205 initially identified records, 26 articles met all inclusion criteria and were included in the final stage (Figure 2).

## 3 Modulation of *P. gingivalis* interactions with other bacterial species by AI-1

AI-1 is predominantly produced by several periodontal bacteria, including species within the genera *Aggregatibacter*, *Fusobacterium*, and *Treponema* (Muras et al. 2020). AI-1 enables bacteria to sense the density, thereby allowing bacterial survival and pathogenicity. The AI-1 signal *N*-acyl homoserine lactone (AHL), was also detected in a multi-species oral biofilms including *Streptococcus oralis*, *Veillonella parvula*, *Actinomyces naeslundii*, *Fusobacterium nucleatum* (*F. nucleatum*), *Aggregatibacter actinomycetemcomitans* (*A. actinomycetemcomitans*) and *P. gingivalis*. In oral polymicrobial biofilms, the presence of AI-1 can enhance the recruitment of *P. gingivalis*, allowing it to take advantage of the protective and nutrient-rich milieu afforded by biofilm formation (9). AI-1 produced by *P. gingivalis* reduced the biofilm formation and metabolic activity of *S. mutans* when these two bacteria were grown together. Thus, the interaction between *P. gingivalis* and *S. mutans* through AI-1 not only reduced the pathogenic potential of *S. mutans* but may also indicated that AI-1 signaling plays a regulatory role between periodontal and cariogenic pathogens (7).

In summary, AI-1, predominantly produced by various periodontal bacteria, plays a crucial role in bacterial communication and survival within oral biofilms. AI-1 facilitates the recruitment and virulence of *P. gingivalis* in multi-species biofilms, enhancing its pathogenic potential. Furthermore, the interaction between *P. gingivalis* and *S. mutans* through AI-1 signaling reduces the pathogenicity of *S. mutans*, suggesting a regulatory role between periodontal and cariogenic pathogens. Understanding AI-1's influence on microbial dynamics and biofilm stability is crucial for grasping the complexities of oral microbial ecology and disease progression.

## 4 Modulation of *P. gingivalis* interactions with other bacterial species by AI-2

Quorum sensing molecule, AI-2, a process that bacteria use to communicate with each other and coordinate their behavior based on population density*.* AI-2 is synthesized by the enzyme LuxS and considered as a universal signaling molecule both in gram-positive and gram-negative bacterial species, making it as a key player in interspecies community (10). By producing and detecting AI-2, *P. gingivalis* can sense the presence of other bacterial cells in its environment and adjust its behavior in interaction with *Streptococcus* species. The *luxS* gene is responsible for producing an AI-2 signaling molecule, which is essential to establish complex biofilm architectures in *P. gingivalis* with *S. mutans* (11). AI-2 facilitates *P. gingivalis* in modifying its behavior to capitalize on the metabolic and structural advantages offered by *S. gordonii* (12). When the LuxS mutant in *S. gordonii* reduced AI-2 production, *S. gordonii* maintained individual growth and biofilm formation but failed to form a cooperative biofilm with *P. gingivalis,* indicting AI-2 favors a cooperative interaction between *S. gordonii* and *P. gingivalis* (13). Furthermore, contact between *S. gordonii* and *P. gingivalis* triggers a signaling cascade that can regulate AI-2 activity (14). In this interaction, the Mfa-Ssp binding event of *S. gordonii* is necessary for initiation of the Ltp1(cytoplasmic eukaryotic-type Low Molecular Weight Tyrosine Phosphatase) -CdhR (LuxR-family transcriptional regulator) pathway of *P. gingivalis*. CdhR of *P. gingivalis* modulates community dynamics by repressing *luxS*, forming a feedback loop with AI-2, which influences the composition and stability of the *P. gingivalis*-*S. gordonii* community (15). Additionally, Ltp1 of *P. gingivalis* plays a crucial role in the downregulation exopolysaccharide, which contributes to maintaining an optimal anaerobic environment favorable for *P. gingivalis*. This regulatory mechanism supports the stability and composition of the *P. gingivalis*-*S. gordonii* community by influencing AI-2-mediated signaling pathways, which are integral to the feedback loop modulating both microbial interaction and community dynamics (16, 17). Overall, the ability of *P. gingivalis* to detect AI-2 allows it to adapt its behavior to exploit the advantages presented by *S. gordonii*, fostering cooperative biofilm formation. By facilitating interactions between *P. gingivalis* and *S. gordonii*, AI-2 enhances the formation of bacterial communities that can significantly shift the balance between health and disease in the oral microbiome. These findings highlight the importance of AI-2 in facilitating interspecies cooperation, emphasizing the complex dynamics of bacterial communities and their reliance on biochemical signaling for survival and interaction.

AI-2 as a crucial interspecies signaling molecule significantly influences the interactions between *P. gingivalis* and other periodontal microbiome within the complex ecosystem (18). *F. nucleatum* is a major coaggregation bridge organism that links early colonising commensals and late pathogenic colonisers in dental biofilms, possesses the ability to co-aggregate with *P. gingivalis*. The external addition of partially purified AI-2 derived from *F. nucleatum* significantly influenced biofilm formation in both monospecific and multispecies cultures, including *P. gingivalis*, *Treponema denticola* (*T. denticola*), and *Tannerella forsythia* (*T. forsythia*). In the presence of AI-2, these biofilms demonstrated increased biomass, greater average depth, and enhanced coaggregation among bacterial species (19). In this synergistic interaction, *F. nucleatum* not only offers a structural framework for *P. gingivalis* adhesion but also contributes vital nutrients that support its growth through AI-2.

The collaborative dynamic established by AI-2 signaling can lead to a heightened inflammatory response, exacerbating periodontal tissue destruction and overall disease severity. This ability to engage in competitive signaling also allows *P. gingivalis* to disrupt the balance of commensal and pathogenic species in the oral cavity, shifting the prevailing microbial community towards a more pathogenic state. AI-2 mediates interactions with additional oral bacteria, such as *Prevotella intermedia* (*P. intermedia*), which are prevalent components of the subgingival microbiota. *P. intermedia* exerted synergistic effects with *P. gingivalis* W83 but antagonistic effects with strain ATCC33277 through AI-2 in regulating the virulence expression of *ribD* and *orpM* (riboflavin metabolism) (20). *Filifactor alocis* (*F. alocis*) is a gram-positive anaerobe that is emerging as an important periodontal pathogen. It interacts variably with other oral pathogens, with its colonization influenced by the presence of *P. gingivalis*, which utilizes AI-2 signaling to modulate community dynamics. While *P. gingivalis* can inhibit *F. alocis* accumulation, it simultaneously benefited from their association, highlighting the complexity of interspecies interactions. Ultimately, the presence of *F. nucleatum* enhanced *F. alocis* accumulation, suggesting that the spatial composition and signaling mechanisms, including AI-2 production, were critical for determining the microbial microenvironments in which *F. alocis* thrived (21). AI-2 of *A. actinomycetemcomitans* affected the neighboring periodontal pathogen, *P. gingivalis*, by modulating the expression of *luxS*-regulated genes, such as *uvrB* and *hasF* which helped *P. gingivalis* repair DNA damage and aided in acquiring iron enabling it to survive under stressful conditions and effectively establish infections in periodontal diseases (22). These multifaceted relationships illustrate how interspecies communication through AI-2 can lead to increased pathogenicity and the advancement of periodontal disease.

The ability of *P. gingivalis* to sense and respond to AI-2 further extends to its influence on establishing the overall composition of the microbial community. By altering its virulence factor production and gene expression profiles in response to AI-2 signaling, *P. gingivalis* can reshape the dynamics of its interactions with various oral bacteria, favoring the establishment of a pathogenic community. This capacity to engage in complex signaling networks through AI-2 highlights the profound impact of interspecies communication on the pathogenicity of *P. gingivalis* in periodontal (Table 1).

**Table 1:** Roles of the signaling molecules in quorum sensing of *P. gingivalis* with other bacteria.

Signaling molecules	Roles	Source [References]
Autoinducer-1	Facilitating formation of symbiotic biofilm between *P. gingivalis* and *S. oralis*, *V. parvula*, *A. naeslundii*, *F. nucleatum*, *A. actinomycetemcomitans*	*S. oralis, V. parvula*, *A. naeslundii, F. nucleatum, A. actinomycetemcomitan*) (9)
	Maintaining microbial balance between *P. gingivalis* and *S. oralis*, *V. parvula*, *A. naeslundii*, *F. nucleatum*, *A. actinomycetemcomitans*	*S. oralis, V. parvula*, *A. naeslundii, F. nucleatum, A. actinomycetemcomitan*) (9)
	Inhibiting biofilm formation of *S. mutans*	*P. gingivalis* (7)
	Reducing metabolic activity of *S. mutans*	*P. gingivalis* (7)
Autoinducer-2	Fostering development of biofilm architectures of *S. mutans*	*P. gingivalis* (11)
	Modifying behavior of *P. gingivalis* to use metabolic and structural advantages provided by *S. gordonii*	*P. gingivalis* (12)
	Inhibiting the accumulation of *F. alocis*	*P. gingivalis* (21)
	Maintaining biofilm formation with *P. gingivalis*	*S. gordonii* (13)
	Enhancing interaction with *P. gingivalis*	*S. gordonii* (14)
	Regulating the community dynamics with *P. gingivalis*	*S. gordonii* (15)
	Constrained the community composition and stability with *P. gingivalis*	*S. gordonii* (16)
	Maintaining an optimal environment for development with *P. gingivalis*	*S. gordonii* (17)
	Enhancing biofilm formation of periodontal microbiome and promoted growth of *P. gingivalis*	*F. nucleatum* (19)
	Regulating virulence factors expression in *P. gingivalis*	*P. intermedia* (20)
	Helping *P. gingivalis* repair DNA damage and aiding in acquiring iron	*A. actinomycetemcomitans* (22)

## 5 Impact of QS inhibitors on *P. gingivalis* with interbacterial interactions

QS inhibitors have shown significant promise in mitigating the pathogenic effects of *P. gingivalis* and its interactions with other bacteria within the oral microbiome. By disrupting the intercellular communication essential for coordinating behaviors in bacterial communities, these inhibitors can impair biofilm formation, virulence factor expression, and the overall dynamics of microbial interactions. The two most studied categories of QS inhibitors are those targeting autoinducer-1 (AI-1) and autoinducer-2 (AI-2), each exhibiting distinct mechanisms of action and effects on *P. gingivalis* and its interbacterial relationships.

### 5.1 AI-1 inhibitors

Inhibitors targeting AI-1 function by disrupting this signaling process, effectively hindering the ability of bacteria to communicate and cooperate. For instance, the addition of acyl-homoserine lactones (AHLs), such as C6-HSL and C12-HSL, in anaerobic dental plaque cultures significantly influenced the microbial community dynamics, promoting the abundance of late colonizing periodontal pathogens like *P. gingivalis*. Specifically, C6-HSL enhanced *Veillonella* abundance while C12-HSL reduced *Fusobacterium* levels (23). The implications of AI-1 inhibition extend beyond individual bacterial interactions; they also affect the structure and composition of biofilms.

Notably, enzymatic inhibitors, such as lactonases, have been characterized as effective agents that degrade the AI-1 signaling molecules, preventing them from reaching their receptors. By hydrolyzing the AHLs, the wide spectrum AHL-lactonase Aii20J effectively dismantled the communication network, leading to decreased expression of virulence factors in *P. gingivalis*. Furthermore, it significantly inhibited oral biofilm formation in different *in vitro* biofilm models and caused important changes in bacterial composition (9). Another two AHL lactonases, SsoPox from the Phosphotriesterase-like Lactonase (PLL) family and GcL from the Metallo β-Lactamase (MLL) family, was reported to change microbial population structures in both planktonic and biofilm states, resulting in the increase in the abundance of commensal and pioneer colonizer species (e.g., *Lactobacillales*, *Streptococcus*, *Actinomyces*) and reduce in the abundance of periodontal pathogens including *P. gingivalis*, *T. forsythia* and *T. denticola* (23).

The potential of active substances was also demonstrated. A recent study reported that a hydrogel combining carvacrol and magnolol significantly reduced pro-inflammatory cytokines in diabetic Wistar rats with periodontitis (24). The carvacrol was verified to competitively block AI-1 pathway by binding with LuxI-type AHL synthases and/or LuxR-type AHL receptor proteins (25). By disrupting QS, AI-1 inhibitors can alter the balance of bacterial species within the biofilm, potentially fostering a switch from a pathogenic to a more beneficial biofilm composition.

### 5.2 AI-2 inhibitors

AI-2 represents a more universal QS signaling molecule, synthesized by a wide range of bacterial species. This characteristic makes AI-2 inhibitors important players in modulating the interactions of *P. gingivalis* with various oral bacterial communities.

D-arabinose significantly reduced the formation of biofilms by single bacteria or consortia of *S. oralis*, *F. nucleatum*, and *P. gingivalis* by inhibiting the activity of AI-2 (26). Similarly, D-galactose markedly inhibited the biofilm formation of *F. nucleatum*, *P. gingivalis*, and *T. forsythia* induced by the AI-2 of *F. nucleatum* without affecting *F. nucleatum* growth (27). Apart from the monosaccharides, the bromofuranone analogs, such as 3-(dibromomethylene)isobenzofuran-1(3H)-one derivatives, also exerted inhibitory activities against biofilm formation of *F. nucleatum*, *P. gingivalis*, and *T. forsythia* (28). Moreover, the combination of (5Z)-4-bromo-5-(bromomethylene)-2(5H)-furanone (furanone compound) and D-ribose inhibited AI-2-induced biofilm formation and coaggregation of single and dual species and coaggregation between *F. nucleatum* and each species of the “red complex” including *P. gingivalis*, *T. forsythia*, and *T. denticola*. The complex also inhibited the expression of the representative adhesion molecules of the periodontopathogens, FadA of *F. nucleatum*, RgpA of *P. gingivalis*, Msp of *T. denticola*, and BspA of *T. forsythia* (19). The *in vivo* effects of the complex were showed in the mice infection model which demonstrated a reduction of bone destruction and a decrease in the number of periodontal bacteria (29, 30).

Azadirachta indica (Neem), a unique and traditional source of antioxidant and antibacterial compounds from India contains catechin. It demonstrated a significant reduction in LuxS activity, with decreases in *P. gingivalis* and *Alcaligenes faecalis*, indicating its capacity to disrupt intercellular communication among biofilm-forming strains (31). Phloretin and its analogs inhibited biofilm formation and intercellular communication mediated by AI-2 in mixed bacterial communities, particularly those involving *P. gingivalis*, *F. nucleatum*, and *S. mitis*. Structural modifications of these flavonoids could enhance their anti-biofilm efficacy by targeting AI-2 production among these pathogenic oral bacteria (32). DMTU (1,3-di-m-tolyl-urea), a biocompatible aromatic compound effectively inhibited and disrupted multispecies oral biofilms composed of *S. gordonii*, *F. nucleatum*, *P. gingivalis*, and *A. actinomycetemcomitans* without exhibiting bactericidal activity. The study also revealed significant downregulation of biofilm and virulence-related genes in *P. gingivalis*, particularly the AI-2 signalling *luxS* gene, in multispecies biofilm contexts, suggesting that multispecies interactions influence gene expression in AI-2 pathway (33). Antimicrobial photodynamic therapy (aPDT) using indocyanine green-loaded nanospheres (ICG-Nano/c) effectively reduces the bacterial load in polymicrobial periodontal biofilms, showcasing significant potential as an alternative or adjunctive treatment to conventional antibiotics. Additionally, the observed decrease in *luxS* expression in both *P. gingivalis* and *S. gordonii* indicated that aPDT may influence QS pathways, further supporting its role as a promising strategy to combat biofilm-related infections and address the increasing health challenges posed by antimicrobial resistance (34).

The application of AI-2 inhibitors disrupts interspecies communication, inhibiting pathogenic species like *P. gingivalis* to grow while simultaneously fostering the recovery of commensal organisms that can suppress its virulence. These inhibitors not only directly target bacterial populations but also enhance therapeutic strategies aimed at restoring a balanced oral microbiome (Table 2). Challenges for QS inhibitors application in clinical trials include variability in patient response, difficulty in achieving effective concentrations at infection sites, and potential side effects. Highlighted gaps include a lack of large-scale human studies and long-term efficacy data. Hence, QS study limitations often involve small sample sizes and single *in vitro* experiments. Despite these challenges, QS inhibitors show clinical potential as adjuncts to conventional periodontal therapies by targeting bacterial communication, reducing virulence, and enhancing treatment outcomes.

**Table 2:** The actions and effects of quorum sensing inhibitors against *P. gingivalis*.

Quorum sensing inhibitor (abbreviation)	Action	Effects [References]
C6- homoserine lactone	Inhibiting Autoinducer-1	Promoting the abundance of late colonizing periodontal pathogens (23)
C12- homoserine lactone		Influencing the microbial community dynamics in anaerobic dental plaque (23)
AHL-lactonase Aii20J	Inhibiting Autoinducer-1	Reducing virulence factor expression of *P. gingivalis*, and inhibiting oral biofilm formation (9)
AHL-lactonase SsoPox	Inhibiting Autoinducer-1	Changing microbial population structures, increasing commensal and pioneer colonizer species abundance, reducing periodontal pathogens abundance (23)
AHL-lactonase GcL		
Carvacrol	Inhibiting Autoinducer-1	Reducing biofilm-induced pro-inflammatory cytokines in periodontitis (24)
D-arabinose	Inhibiting Autoinducer-2	Reducing the formation of biofilms by single bacteria or consortia of *S. oralis*, *F. nucleatum*, and *P. gingivalis* (26)
D-galactose	Inhibiting Autoinducer-2	Inhibiting the biofilm formation of *F. nucleatum*, *P. gingivalis*, and *T. forsythia* without affecting bacterial growth (27)
D-ribose	Inhibiting Autoinducer-2	Decreasing *P. gingivalis* population and reducing inflammation in periodontal tissues (29, 30)
Bromofuranone	Inhibiting Autoinducer-2	Inhibiting biofilm formation by *F. nucleatum*, *P. gingivalis*, and *T. forsythia* (28)
Azadirachta indica	Inhibiting Autoinducer-2	Decreasing *P. gingivalis* and *Alcaligenes faecalis* populations (31)
Phloretin	Inhibiting Autoinducer-2	Reducing biomass in mixed flora destructively affecting *P. gingivalis* biofilms (32)
1,3-di-meta-tolylurea	Inhibiting Autoinducer-2	Inhibiting multispecies biofilms of *S. gordonii*, *F. nucleatum*, *P. gingivalis*, and *A. actinomycetemcomitans* (33)
Indocyanine green-loaded nanospheres	Inhibiting Autoinducer-2	Reducing bacterial load in periodontal biofilms (34)

## 6 Conclusion

In conclusion, the interaction between *P. gingivalis* and oral microbial community highlights the significance of QS as a critical regulatory mechanism in microbial communication and ecological dynamics. Through AI-1/AI-2 signaling, *P. gingivalis* not only enhances its virulence and promotes biofilm persistence but also drives dysbiosis and reshapes microenvironments. QS inhibitors present a promising therapeutic strategy to rebalance microbial communities rather than eradicate pathogens. Combination of QS inhibitors with conventional periodontal treatments may offer innovative and microbiome-preserving approaches to manage periodontal diseases.

## Data Availability

The raw data supporting the conclusions of this article will be made available by the authors, without undue reservation.
